# Vps4a Regulates Autophagic Flux to Prevent Hypertrophic Cardiomyopathy

**DOI:** 10.3390/ijms241310800

**Published:** 2023-06-28

**Authors:** Xiaozhi Huang, Jiayin Zhang, Wenyi Wang, Zhishan Huang, Peidong Han

**Affiliations:** 1 Division of Medical Genetics and Genomics, The Children’s Hospital, Zhejiang University School of Medicine, National Clinical Research Center for Child Health, Hangzhou 310058, China; 2 Institute of Genetics, Zhejiang University School of Medicine, Hangzhou 310058, China; 3 Zhejiang Provincial Key Lab of Genetic and Developmental Disorder, Hangzhou 310058, China

**Keywords:** ESCRT, Vps4a, autophagy, heart, heart failure

## Abstract

Autophagy has stabilizing functions for cardiomyocytes. Recent studies indicate that an impairment in the autophagy pathway can seriously affect morphology and function, potentially leading to heart failure. However, the role and the underlying mechanism of the endosomal sorting complex required for transport (ESCRT) family protein, in particular the AAA-ATPase vacuolar protein sorting 4a (Vps4a), in regulating myocardial autophagy remains unclear. In the present study, cardiomyocyte-specific Vps4a knockout mice were generated by crossing *Vps4a^flox/flox^* (*Vps4a^fl/fl^*) with *Myh6-cre* transgenic mice. As a result, we observed a partially dilated left ventricular (LV) chamber, a significant increase in heart weight to body weight ratio (HW/BW), and heart weight to tibial length ratio (HW/TL), hypertrophic cardiomyopathy and early lethality starting at 3 months of age. Hematoxylin-eosin (HE), immunofluorescence assay (IFA), and Western blot (WB) revealed autophagosome accumulation in cardiomyocytes. A transcriptome-based analysis and autophagic flux tracking by AAV-RFP-GFP-LC3 showed that the autophagic flux was blocked in Vps4a knockout cardiomyocytes. In addition, we provided in vitro evidence demonstrating that Vps4a and LC3 were partially co-localized in cardiomyocytes, and the knockdown of Vps4a led to the accumulation of autophagosomes in cardiomyocytes. Similarly, the transfection of cardiomyocytes with adenovirus (Adv) mCherry-GFP-LC3 further indicated that the autophagic flux was blocked in cells with deficient levels of Vps4a. Finally, an electron microscope (EM) showed that the compromised sealing of autophagosome blocked the autophagic flux in Vps4a-depleted cardiomyocytes. These findings revealed that Vps4a contributed to the sealing of autophagosomes in cardiomyocytes. Therefore, we demonstrated that Vps4a deletion could block the autophagic flux, leading to the accumulation of degradation substances and compromised cardiac function. Overall, this study provides insights into a new theoretical basis for which autophagy may represent a therapeutic target for cardiovascular diseases.

## 1. Introduction

Autophagy is a common cellular catabolic process in which harmful intracellular components are degraded [1,2]. During this process, the cytoplasmic materials are sequestered within the autophagosome and then fused with the lysosome to form autophagolysosome, which is finally degraded to maintain cellular homeostasis [2]. Therefore, autophagy contributes to many physiological and pathological processes, such as differentiation, development, and adaption of the cell to an adverse environment [3,4].

In the heart, autophagy also plays an important role in maintaining the homeostasis of various types of cells, such as cardiomyocytes, endothelial cells, and arterial smooth muscle cells. In physiological conditions, the impairment of the autophagic flux through pharmacological or genetic approaches causes cardiovascular diseases, such as myocardial infarction, cardiomyopathies, and atherosclerosis [5,6]. In this regard, cardiac-specific Atg5 (autophagy-related 5) knockout mice showed abnormal cardiac function, characterized by the disorganized sarcomere, mitochondrial misalignment and aggregation, and cardiac hypertrophy [7,8]. In pathological conditions, cardiovascular diseases such as myocardial infarction, cardiac hypertrophy, and various types of cardiomyopathies are also accompanied by changes in the level of autophagy, which plays different functions. In myocardial ischemia, induction of autophagy is protective, whereas reperfusion stimulates autophagy and is implicated in causing cell death [9]. Ischemia/reperfusion injury impairs autophagosome clearance mediated by declination lysosome-associated membrane protein-2 and upregulation of BECLIN-1, contributing to increased cardiomyocyte death [10]. However, transcription factor EB (TFEB) reprograms macrophage lysosomal lipid metabolism and will attenuate remodeling after I/R [11]. Trehalose (TRE) also induces the activation of autophagy and improves cardiac remodeling after myocardial infarction [12]. Furthermore, mTORC1 is an important negative regulator of autophagy [13]. In cardiac hypertrophy, oxidation of PKG1 α controls mTORC1 activation and decreases the level of autophagy-influenced cardiac function [13,14]. The studies showing that protein kinase G will alleviate cardiac disease have been confirmed [15]. In the pathogenesis of load-induced heart failure, Beclin 1 decreased cardiomyocyte autophagy and diminished pathological remodeling. Conversely, Beclin 1 overexpression heightened autophagic activity and accentuated pathological remodeling [16]. Therefore, autophagy is an adaptive response to an adverse environment in cardiac disease [14,17].

In mammalian cells, the membrane remodeling events such as budding, fusion, and fission are regulated by the endosomal sorting complex required for transport (ESCRT), which includes ESCRT-0, ESCRT-I, ESCRT-II, ESCRT-III, and the AAA-ATPase vacuolar protein sorting 4a (Vps4a) [18,19]. This process plays an essential role in regulating different cellular processes, such as microvesicles and exosome biogenesis, membrane repair, neuron pruning, and virus budding [19,20]. Autophagy includes a variety of membrane remodeling, including phagophore initiation, autophagosome formation, and autophagosome-lysosome fusion, which requires the involvement of ESCRT family proteins [21,22,23,24,25]. For example, the downregulation of charged multivesicular body protein 2A (CHMP2A) in mammalian cells [26], ist1 factors (Ist1) associated with ESCRT-III in neurons [27], and Vps4 in the axon of drosophila [28] can impair the autophagic flux leading to the disease onset. SKD1 AAA-ATPase, another name for Vps4a, is involved in autolysosome formation in vitro [29]. However, to what extent Vps4a can regulate autophagic flux in cardiomyocytes remains unknown. As a result, the aim of the present study was to investigate the mechanism and the role of Vps4a in cardiac function.

## 2. Results

### 2.1. Mice with a Conditional Vps4a Knockout Developed Heart Failure and Showed Increased Mortality

**Figure 1:**
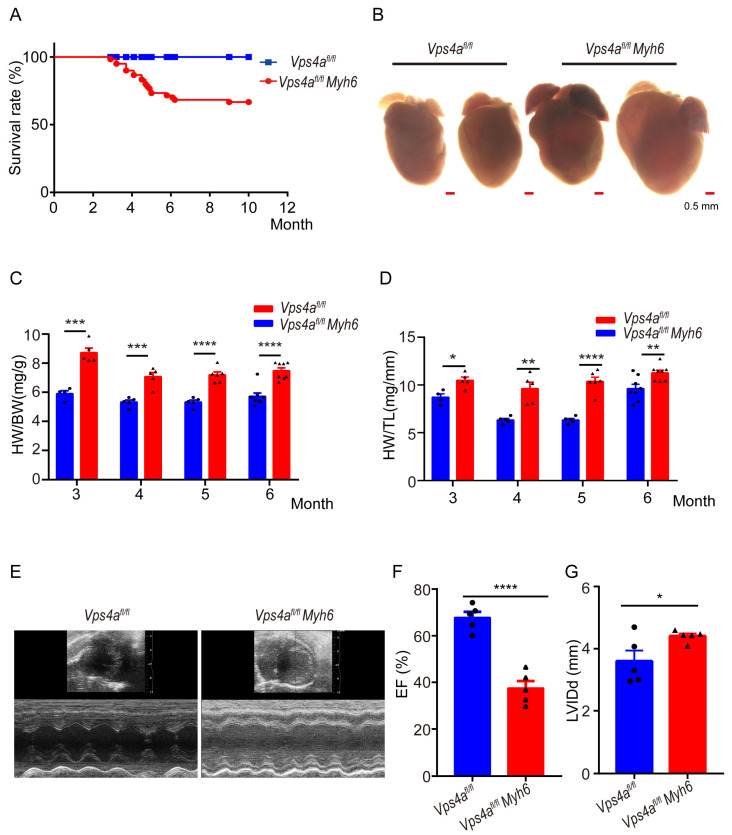
Cardiomyocyte-specific Vps4a deletion affects the anatomy and function of the heart. (**A**) Kaplan–Meyer curve of *Vps4a^fl/fl^ Myh6* and *Vps4a^fl/fl^* mice (n = 60). (**B**) Bright-field images of the heart from *Vps4a^fl/fl^* and *Vps4a^fl/fl^ Myh6* mice. (**C**,**D**) Statistical analysis of HW/BW and HW/TL in *Vps4a^fl/fl^* and *Vps4a^fl/fl^ Myh6* mice between 3 to 6 months of age (n = 4–8 per group). (**E**–**G**) Representative transthoracic echocardiography images and statistics of ejection fraction (EF) and left ventricular internal dimension (LVIDd) in *Vps4a^fl/fl^* and *Vps4a^fl/fl^ Myh6* mice at 4-months old (n = 5). * *p* < 0.05, ** *p* < 0.01, *** *p* < 0.001, **** *p* < 0.0001. Significant differences between groups as determined by a two-tailed paired Student’s *t*-test. Data are expressed as mean and s.e.m. Scale bars: (**B**) 0.5 mm.

To investigate the role of Vps4a for cardiac function, cardiomyocyte-specific mice were generated by crossing *Vps4a^fl/fl^* with *Myh6-cre* transgenic mice. *Vps4a^fl/fl^Myh6-cre* mice were grossly normal at 2 months old. However, starting from 3 months old, partial lethality was observed in Vps4a knockout mice, while some mice were able to survive for more than 12 months (Figure 1A). Vps4a knockout mice displayed conspicuous cardiac hypertrophy at 4 months (Figure 1B). Quantitative analysis showed a marked increase in the heart weight to body weight ratio (HW/BW) and heart weight to tibial length ratio (HW/TL) in Vps4a knockout compared to *Vps4a^fl/fl^* mice (Figure 1C,D). At the age of 4 months old, transthoracic echocardiography also showed a significant decrease in ejection fraction and left ventricular internal dimension end-diastole in Vps4a-deleted hearts (Figure 1E–G and Table S1). Together, these data suggested that cardiomyocyte-specific Vps4a knockout mice developed a progressive cardiac dysfunction which eventually led to heart failure.

### 2.2. Histology Showed Many Vesicles in the Cardiomyocytes of Knockout Mice

**Figure 2:**
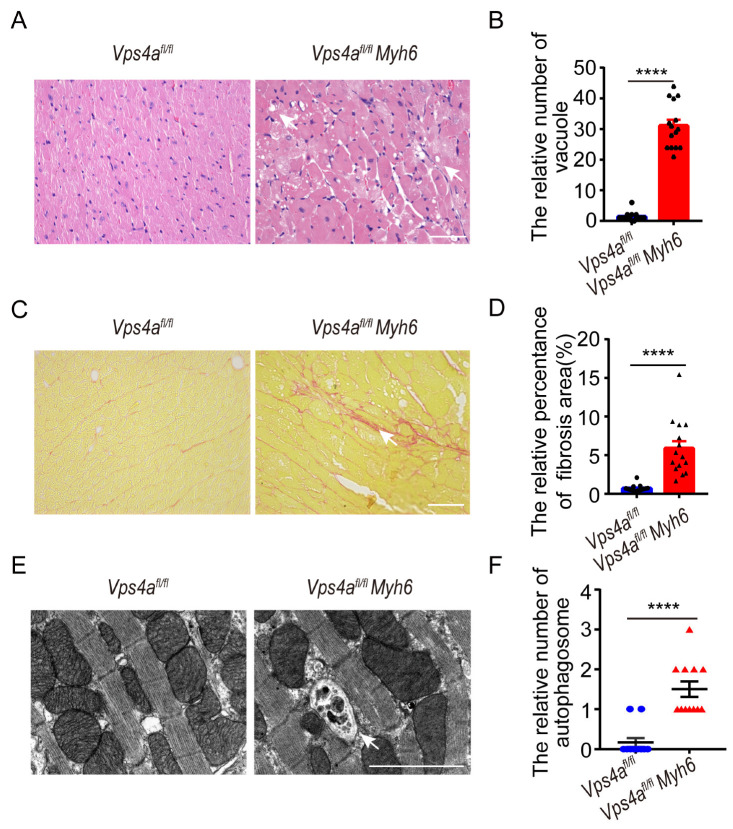
Vps4a-depleted cardiomyocytes showed vacuoles, autophagosomes, and fibrosis. (**A**) The heart sections of *Vps4a^fl/fl^* and *Vps4a^fl/fl^ Myh6* knockout mice were subjected to H&E. Arrows indicate vacuoles in the cardiomyocytes. (**B**) Statistical analysis number of vacuoles in *Vps4a^fl/fl^* and *Vps4a^fl/fl^ Myh6* mice at 3 months of age (n = 3 per group); images in 15 randomly selected visual fields were counted. (**C**) The heart sections of *Vps4a^fl/fl^* and *Vps4a ^fl/fl^ Myh6* knockout mice were subjected to Sirius Red staining. Arrow indicate area of fibrotic cardiomyocytes. (**D**) Statistical analysis area of fibrosis in *Vps4a^fl/fl^* and *Vps4a^fl/fl^ Myh6* mice at 3 months of age (n = 3 per group); images in 15 randomly selected visual fields were counted. (**E**) The images of autophagosome in *Vps4a^fl/fl^ Myh6* knockout mice were observed by electron microscope. Arrow indicate autophagosome in the cardiomyocytes. (**F**) Statistical analysis number of autophagosomes in *Vps4a^fl/fl^ Myh6* knockout mice (n = 3 per group). Images in 10 randomly selected visual fields were counted. **** *p* < 0.0001. Significant differences between groups as determined by a two-tailed paired Student’s *t*-test. Data are expressed as mean and s.e.m. Scale bars: (**A**,**C**) 50 μm, (**E**) 2 μm.

To further investigate the cause of death in cardiomyocyte-specific Vps4a knockout mice, the heart tissues from 3-month-old *Vps4a^fl/fl^* and *Vps4a^fl/fl^ Myh6* mice were examined by HE staining. As a result, we observed that there were a large number of vacuoles in the cardiomyocytes of Vps4a-deleted heart tissues (Figure 2A,B, arrows). Vacuolated and fibrotic cardiomyocytes were observed in Vps4a knockout heart tissues by Sirius Red staining (Figure 2C,D, arrow). To further identify the vesicular structures causing the vacuoles, electron microscope analysis was employed, which revealed large autophagosomes in *Vps4a^fl/fl^ Myh6* heart tissues that were not subjected to degradation (Figure 2E,F and Figure S1 arrow). These results suggested that Vps4a may affect the autophagy process in cardiomyocytes.

### 2.3. RNA Sequencing Revealed That Autophagy Pathway Was Impaired in Vps4a Knockout Mice

**Figure 3:**
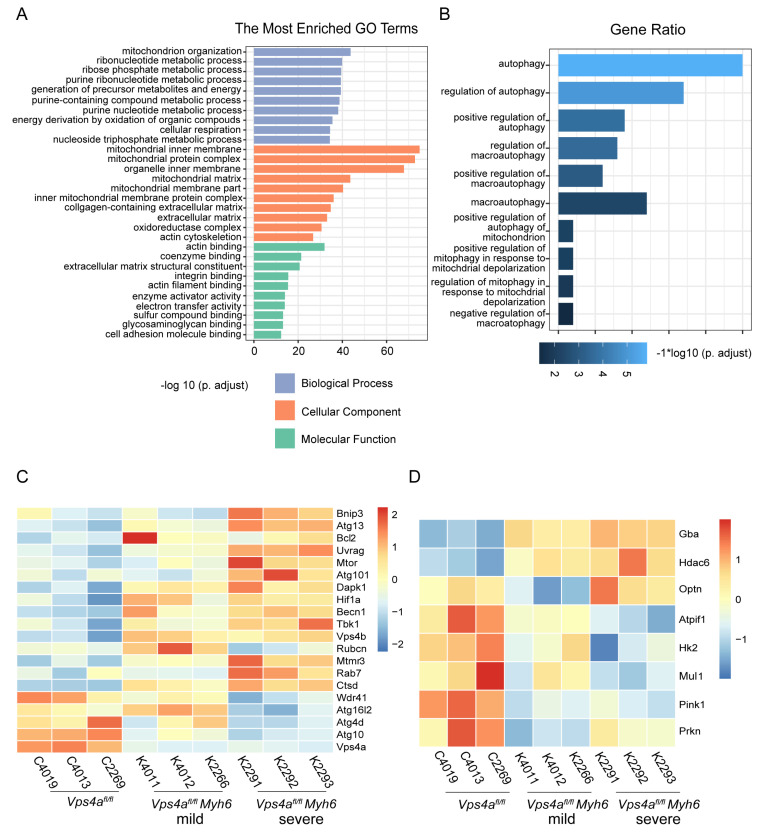
RNA sequencing revealed that autophagy pathway was impaired in Vps4a knockout mice. (**A**) GO analysis of differentially expressed genes in heart samples of *Vps4a^fl/fl^* and *Vps4a^fl/fl^ Myh6* mice, with the top 10 enriched GO terms observed in biological process, cellular component, and molecular function. (**B**) GO analysis of differentially expressed genes in heart samples from *Vps4a^fl/fl^* and *Vps4a^fl/fl^ Myh6* mice, showing GO terms related to autophagy. (**C**,**D**) Heat map analysis of differential expression of genes associated with autophagy and mitophagy in the hearts from *Vps4a^fl/fl^* and *Vps4a^fl/fl^ Myh6* mice. (Applying the combined criteria of *q* ≤ 0.05 and fold change ≥1.3, *p* < 0.05, Bonferroni corrected), (*Vps4a^fl/fl^* = 3, *Vps4a^fl/fl^ Myh6* = 6, left: (mild), right: (severe)).

To further interrogate the effect of Vps4a knockout on the cardiomyocyte function, RNA-Sequencing on *Vps4a^fl/fl^ Myh6* and *Vps4a^fl/fl^* murine heart tissues was performed. Particularly, *Vps4a^fl/fl^ Myh6* mice were separated into mild and severe groups based on phenotypical analysis. By analyzing biological process (BP), cellular component (CC), and molecular function (MF), we found that the differentially expressed genes in *Vps4a^fl/fl^* and *Vps4a^fl/fl^ Myh6* mice were enriched in mitochondrion organization, mitochondrial inner membrane, and organelle inner membrane (Figure 3A). This result is consistent with the role of ESCRT family proteins in the remodeling of the plasma membrane and inner membranes. Intriguingly, we also found enrichment in genes that were related to autophagy in Vps4a knockout hearts (Figure 3B). Heat map analysis demonstrated that the expression of early autophagosomes initiation genes such as Atg13, Bcl2, and Becn1 was significantly increased in *Vps4a^fl/fl^ Myh6* mice, especially in *Vps4a^fl/fl^ Myh6* (severe) group (Figure 3C and Table S2). Simultaneously, the expression level of mitophagy-related gene pink1 was decreased (Figure 3D and Table S3), suggesting that Vps4a loss-of-function in cardiomyocytes is associated with the impairment of autophagic flux as well as mitophagy.

### 2.4. Impaired Autophagic Flux in Vps4a Knockout Mice Leads to Heart Failure

**Figure 4:**
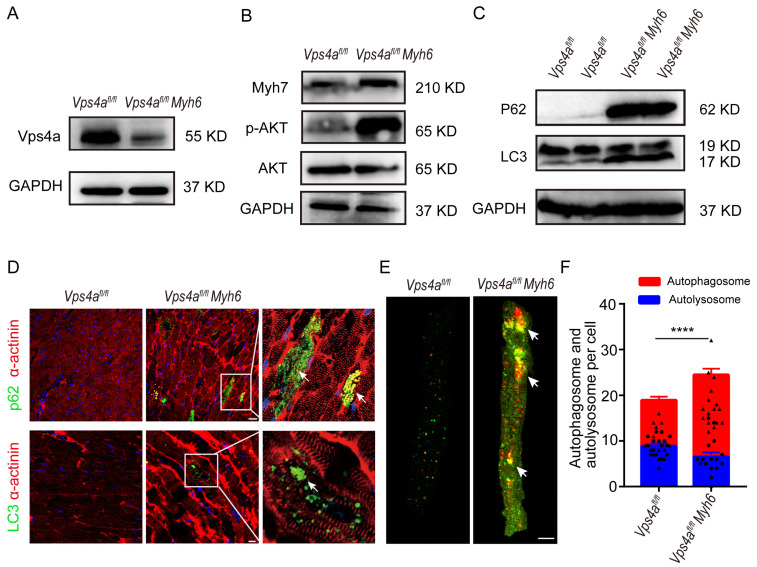
Impaired autophagic flux in Vps4a knockout mice led to abnormal cardiac function. (**A**,**B**) Protein expression levels of Vps4a, Myh7, p-AKT, and AKT in heart samples of *Vps4a^fl/fl^* and *Vps4a^fl/fl^ Myh6* mice analyzed by Western blot. (**C**) Protein expression levels of p62 and LC3 in heart samples of *Vps4a^fl/fl^* and *Vps4a^fl/fl^ Myh6* mice analyzed by Western blot. (**D**) Immunofluorescence assay staining of p62, LC3, and α-actinin in heart sections of *Vps4a^fl/fl^* and *Vps4a^fl/fl^ Myh6* mice. Arrows indicate staining of p62 and LC3. (**E**) Autophagic flux tracked by AAV-RFP-GFP-LC3 injected in *Vps4a^fl/fl^* and *Vps4a^fl/fl^ Myh6* mice. Arrows indicate autophagosome in the cardiomyocytes. (**F**) Statistical analysis of autophagosome (yellow) and autolysosome (red) in cardiomyocytes from *Vps4a^fl/fl^* and *Vps4a^fl/fl^ Myh6* mice (n = 3 per group) in each experiment, 16 cells were counted. **** *p* < 0.0001. Significant differences between groups as determined by a two-tailed paired Student’s *t*-test. Data are expressed as mean and s.e.m. Scale bars: (**D**, top) 20 μm, (**D**, bottom, **E**) 10 μm.

We further determined the effect of Vps4a deletion on the autophagic flux in cardiomyocytes. In 3 months old mice, Western blot analysis showed that Vps4a protein levels were significantly decreased in *Vps4a^fl/fl^ Myh6* mice compared to *Vps4a^fl/fl^* mice, while the expressions of markers for heart failure, such as p-AKT and Myh7, increased (Figure 4A,B and Figure S2A–D). In addition, the levels of autophagy-related genes P62 and LC3 were also significantly increased (Figure 4C and Figure S2E,F). The accumulation of LC3 and P62 in Vps4a knocked out cardiomyocytes was further examined by immunofluorescence staining on tissue sections (Figure 4D). To further assess the impairment of autophagic flux, AAV-RFP-GFP-LC3 was injected into *Vps4a^fl/fl^* and *Vps4a^fl/fl^ Myh6* mice via the tail vein to track the autophagic flux within cardiomyocytes. We found that in *Vps4a^fl/fl^ Myh6* cardiomyocytes, the numbers of autophagosomes (yellow) increased and autolysosomes (red) decreased, and autophagosomes were larger and irregular compared with *Vps4a^fl/fl^* (Figure 4E,F). Taken together, these findings suggested that the autophagic flux in Vps4a-deleted cardiomyocytes was impaired, which led to cardiac dysfunction.

### 2.5. Knockdown of Vps4a Resulted in Compromised Sealing of Autophagosome Which Impaired the Autophagic Flux

**Figure 5:**
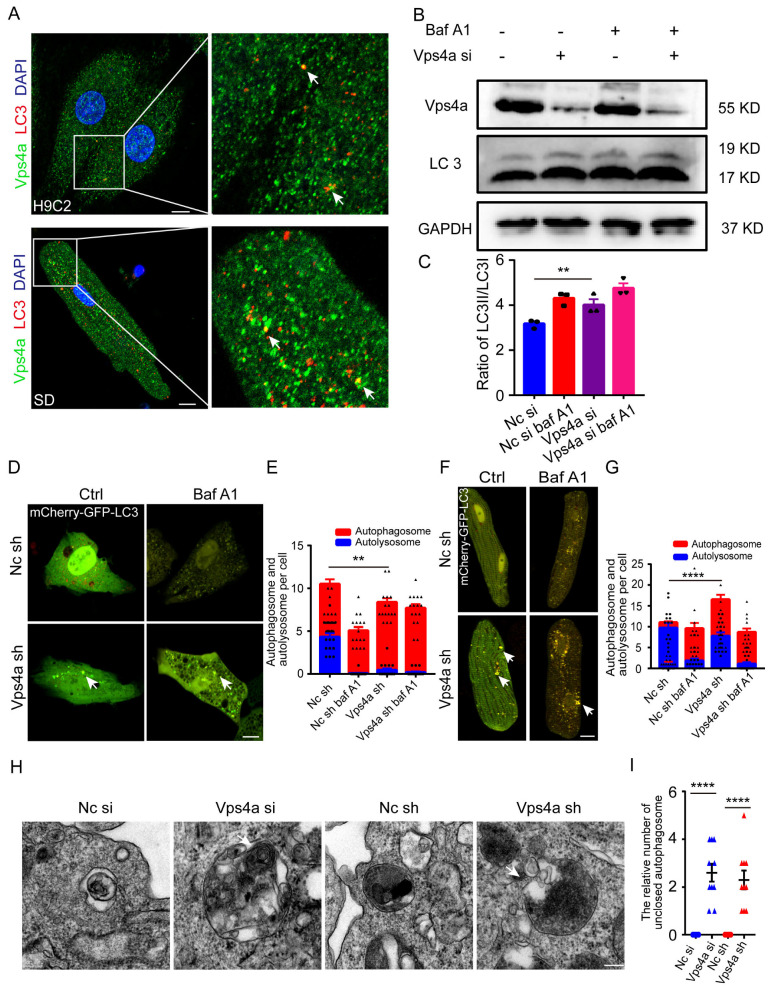
Vps4a is involved in the sealing of autophagosomes within cardiomyocytes and affects the autophagic flux in vitro. (**A**) Immunofluorescence assay staining of Vps4a and LC3 in H9C2 (**top**) and primary cardiomyocytes (**bottom**), with Vps4a and LC3 partially co-localized (arrows). (**B**) Representative Western blot showing LC3 expression levels in H9C2 cells transfected with Vps4a RNA si and Nc RNA si, with increased expression levels of LC3 II in Vps4a knockdown H9C2 cells. (**C**), Western blot analysis was performed to measure LC3II/LC3I protein expression. ** *p <* 0.01 Significant differences between groups as determined by a two-tailed paired Student’s *t*-test in three independent experiments. (**D**) Autophagic flux tracked by Adv-mCherry-GFP-LC3 transfected into H9C2 cells. Arrows indicate autophagosome in the cardiomyocytes. (**E**) Statistic of autophagosome (yellow) and autolysosome (red) in (**C**), (n = 16) ** *p <* 0.01. Significant differences between groups as determined by two-way ANOVA with Tukey’s multiple comparisons test. (**F**) Adv-mCherry-GFP-LC3 was transfected into control and Vps4a knockdown primary cardiomyocytes. Arrows indicate autophagosome in the cardiomyocytes. (**G**) Statistic of autophagosome (yellow) and autolysosome (red) in (**E**), (n = 16) **** *p* < 0.0001. Significant differences between groups as determined by two-way ANOVA with Tukey’s multiple comparisons test. (**H**) Autophagosome observed by electron microscope in H9C2 cells transfected with Vps4a RNA si and Vps4a RNA sh, showing an incomplete autophagosome atresia. Arrow indicate unclosed autophagosome in the cardiomyocytes. (**I**) Statistical analysis number of unclosed autophagosome in Vps4a knockdown H9C2 cells. Images in 10 randomly selected visual fields were counted. **** *p <* 0.0001. Significant differences between groups as determined by a two-tailed paired Student’s *t*-test. Data are expressed as mean and s.e.m. Scale bars: (**A**,**D**,**F**) 10 μm, (**H**) 1 μm.

The involvement of Vps4a in autophagy and its co-localization with LC3 in cardiomyocytes was investigated by immunofluorescence assay (Figure 5A). Upon small molecule inhibitor bafilomycin A1 (Baf A1) treatment, the fusion between phagosome and lysosomes was blocked. As a result, we observed an increase in the expression level of LC3 protein (Figure 5B,C). When the expression of Vps4a was knocked down by siRNA in H9C2 cells, an increase in LC3 II levels was detected by Western blot (Figure 5B), further supporting the role of Vps4a in the autophagic flux. Similarly, tracking Vps4a knockdown cardiomyocytes using Adv-mCherry-GFP-LC3 revealed an increase in the number of autophagosomes which became enlarged and irregular (yellow), while the number of autolysosomes was reduced (red) in H9C2 (Figure 5D,E) and primary cardiomyocytes (Figure 5F,G), consistent with the results obtained by the in vivo experiments. Therefore, Vps4a loss-of-function led to incomplete closure of the autophagosome, resulting in its enlargement and maintenance of an open state as observed by electron microscope, indicating the failure of the membrane fusion in Vps4a knockdown H9C2 cardiomyocytes (Figure 5H,I).

**Figure 6:**
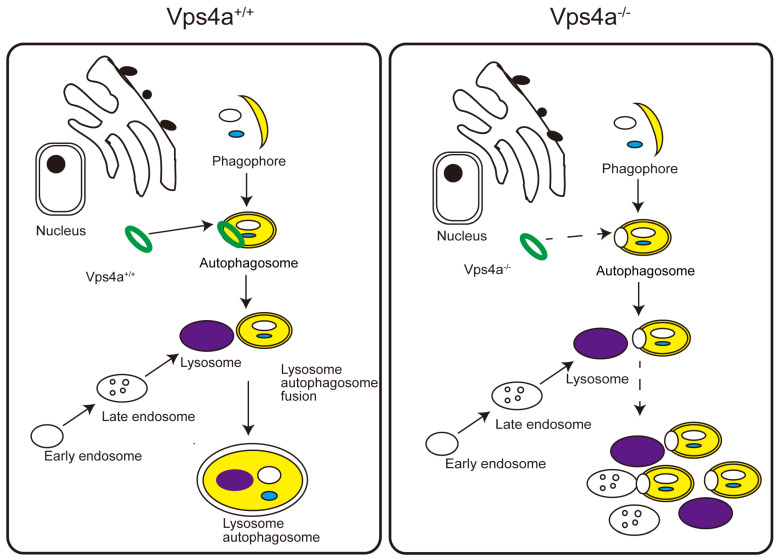
Schematic showing that Vps4a controls autophagic flux to regulate cardiac function. During the process of autophagosome formation, Vps4a is involved in the sealing of the autophagosome. When Vps4a is conditionally deleted in a mouse model, the autophagic flux in cardiomyocytes is blocked, which impairs the degradation of intracellular components and leads to cardiomyocyte death and fibrosis. The reduced cardiac function and heart failure results in early lethality in Vps4a knockout mice.

In conclusion, we found that Vps4a is mainly involved in the sealing of autophagosome in cardiomyocytes and that the Vps4a deletion blocked the autophagic flux, leading to the accumulation of degradation products and impairment of the physiological function of the heart (Figure 6).

## 3. Discussion

Autophagy maintains cellular homeostasis in different types of cells, including cardiomyocytes, endothelial cells, and arterial smooth muscle cells, which are essential for the physiological function of the heart [5]. Multiple cellular abnormalities were found in mice with conditional knockout of the autophagy-related gene ATG7, including disorder of membrane structure, deformation of mitochondria, and accumulation of ubiquitin-positive aggregates [30]. ATG7 variants in humans were associated with several developmental disorders affecting the brain, muscle, and endocrine organs [31]. The ESCRT system is a molecular mechanism involved in the membrane remodeling of intracellular organelles, and it is involved in a variety of cellular functions, including cell division, virus fusion, autophagosome formation, and membrane repair [19,20]. In particular, it has been reported that the ESCRT family protein SKD1 influences the process of autophagy in vitro [29] and subsequent studies reported that Vps4 loss-of-function caused neurological degeneration by affecting the autophagic flux in the Drosophila model [28]. However, the mechanism by which Vps4a affects the autophagic flux in the heart remains elusive. The present study demonstrates that Vps4a deletion leads to partial lethality at about 3 months of age with cardiac dysfunction and hypertrophy. Mechanistically, Vps4a controls autophagic flux in cardiomyocytes by regulating the sealing of autophagosomes. In physiological conditions, a low level of autophagy maintains the normal operation of cardiomyocytes. Once the autophagic flux was blocked by knockout Vps4a, accumulation of ubiquitinated proteins, vacuole formation, myofibrillar disarray, and enhanced intermuscular fibrosis led to contractile dysfunction, cardiac hypertrophy, and heart failure.

Vps4b, which is another key ESCRT family protein, is structurally and functionally similar to Vps4a, being involved in the depolymerization of ESCRT III. In tumor cells, knockout of Vps4b led to an increased expression of Vps4a to compensate for these functions [32,33]. The RNA sequencing results from this study also showed that Vps4b expression was increased in Vps4a knockout mice (Figure 3C). Interestingly, we only observed the lethality in some of the cardiomyocyte-specific Vps4a knockout mice. The fact that other mice did not die shows what the process of sealing autophagosomes can accomplish. There is synthetic lethality between Vps4a and Vps4b [32,33]. Knockdown of Vps4a alone was not sufficient to cause a complete loss of membrane remodeling mediated by ESCRT. Hence, whether this phenotype is associated with the compensatory effect of Vps4b elevation needs to be further investigated.

Several conditional knockouts of autophagy-related genes of the heart have been reported [5]. In this regard, during the cardiomyocyte-specific knockout of ATG5 implemented to block the autophagy pathway in the mouse model, the mice developed left ventricular dilation, systolic dysfunction, and cardiac hypertrophy and incurred death at about 10 months. In addition, cardiomyocytes in this animal model displayed sarcomeric structure disorder, mitochondrial disarrangement, and aggregation [7,8]. Similarly, deletion of Vps4a in the heart also led to left ventricular dilation, decreased systolic function, cardiac hypertrophy, impaired cardiomyocyte structure with vesicular accumulation, and compromised mitochondrial arrangement. The autophagy tracking and electron microscope data further indicated that Vps4a was involved in the closure of autophagosome and therefore affected the autophagic flux. Meanwhile, an electron microscope revealed disorganized intracellular vesicles in Vps4a knockout mice. Notably, Vps4a knockout mice died before ATG5 knockout mice, suggesting that Vps4a may also play an important role in other biological functions in cardiomyocytes. The RNA-sequencing analysis also showed that Vps4a affected mitophagy, with the mitochondria function also severely impaired in Vps4a knockout heart tissues. Nevertheless, how Vps4a controls mitophagy and other forms of vesicle formation in cardiomyocytes requires further investigation.

Cardiovascular diseases such as myocardial infarction, cardiac hypertrophy, and various types of cardiomyopathies are also accompanied by changes in the level of autophagy, which plays different functions. In the ischemia/reperfusion mice model, ischemia stimulates autophagy through an AMPK-dependent mechanism, whereas ischemia/reperfusion stimulates autophagy through a Beclin1-dependent but AMPK-independent mechanism [9]. Therefore, the inactivation of Rheb and inhibition of mTORC1 may represent therapeutic targets to reduce myocardial damage during ischemia [34]. LAPTM4B, also targeted in the modulation of the mTORC1/TFEB pathway, contributes to myocardial I/R-induced impairment of autophagic flux [35]. mTORC1 activation also is indispensable for the development of adaptive cardiac hypertrophy [13]. Oxidation of PKG1α controls mTORC1 activation and depresses autophagy resulting in cardiac hypertrophy. Therefore, targeting protein kinase G regulation of proteostasis will alleviate cardiac disease [14,15]. Overall, autophagy maintains cardiovascular homeostasis in physiological and pathological settings.

In conclusion, Vps4a deficiency results in cardiac dysfunction by impairing the process of autophagic flux, and our findings provide new insight into the development of new therapeutic approaches targeting autophagy for the treatment of cardiovascular diseases.

## 4. Materials and Methods

### 4.1. Animals

*Vps4a^fl/fl^* mice were generated using the CRISPR/Cas9 technology by Biocytogen Pharmaceuticals (Beijing, China). Briefly, to generate *Vps4a^fl/^^fl^*, a targeting vector containing loxP sequences was constructed and co-injected with sgRNAs to produce floxed alleles in intron 3 and intron 5. *Myh6 Cre* mice were purchased from the Jackson Laboratory. *Vps4a^fl/fl^ Myh6* were generated by crossing *Vps4a^fl/fl^* with *Myh6-cre* transgenic mice. We did not observe conspicuous abnormality in the physical activities in *Myh6 Cre* mice in our study, in sharp contrast to Vps4a knockout mice. That is why we did not include *Myh6 Cre* mice as the control. Sprague Dawley rats were purchased from the Zhejiang Academy of Medical Sciences. Procedures were approved by the Animal Care and Use Committee of the University School of Medicine of Zhejiang, China.

### 4.2. Isolation and Culture of Cardiomyocytes

Primary cardiomyocytes were isolated as previously described [36]. Briefly, adult rats (200–250 g) were anesthetized by intraperitoneal injection using pentobarbital sodium (dose 40 mg/kg). The hearts were cannulated onto a Langendorff apparatus and perfused with Tyrode’s solution (mM: NaCl 137, KCl 5.4, HEPES 10, MgCl_2_ 1.2, NaHPO_4_ 1.2, Glucose 10, and Taurine 10) containing collagenase II (Worthington, Vassar, NY, USA, LS004176) and protease (Sigma, St. Louis, MO, USA, P5147). The ventricles were manually dissociated to obtain single cells of cardiomyocytes, which were then plated on culture dishes coated with laminin (Sigma, L2020) for 2 h. Subsequently, cardiomyocytes were cultured in M199 medium (Sigma, M3769) containing 5 mM creatine, 2 mM L-carnitine, 5 mM taurine, 26 mM NaHCO_3_, and 25 mM HEPES (Sigma, H3375). H9C2 cardiomyocyte cells were purchased from the National Collection of Authenticated Cell Cultures and cultured in 100 cm^2^ dishes with DMEM medium at 10% of fetal bovine serum (FBS) and 1% penicillin and streptomycin.

### 4.3. siRNA and shRNA Transfection

siRNAs were obtained from Gene Pharma (Shanghai, China). Specifically, H9C2 cells and primary cardiomyocytes were transfected with 100 nM of siRNA with lipofectamine RNAi MAX (Invitrogen, Waltham, MA, USA, 13778150) for 48 h. Adenoviruses encoding shRNAs were generated using BLOCK-iT Adenoviral RNAi Expression System (Invitrogen, K494100) according to the manufacturer’s protocol.

The following siRNAs and shRNA were used in this study:

*Vps4a* siRNA: GCUACUCAGGAGCAGAUAUTT,

*Control* siRNA: UUCUCCGAACGUGUCACGUTT,

*Vps4a* shRNA: GCTACTCAGGAGCAGATATTT,

*Control* shRNA: TTCTCCGAACGTGTCACGTTT.

### 4.4. Adenovirus and AAV9 Construction and Delivery

Adv-mCherry-GFP-LC3 was kindly provided by Dr. Huang-Tian Yang. Briefly, H9C2 cells and primary cardiomyocytes were incubated with adenovirus for 6 h at an MOI of 250, followed by 48 h of incubation in a standard culture medium. As for in vivo studies, AAV9-RFP-GFP-LC3 was obtained from HANBI Technology (Shanghai, China) and injected into the tail vein of 4-6 weeks-old *Vps4a^fl/fl^ myh6* mice. The images were viewed with Niikon C2 plus (Nikon, Tokyo, Japan) confocal microscope. The number of GFP and RFP dots was determined by manual counting of fluorescent puncta [35].

### 4.5. Protein Isolation and Immunoblotting

Total proteins were extracted from H9C2 cells or cardiac tissues using RIPA buffer (Solarbio, Beijing, China, R0020) supplemented with protease and phosphatase inhibitors (Sangon Biotech, Shanghai, China, C600387, and C500017, respectively). The following antibodies were used in this study: anti-Vps4a (Sigma, SAB4200215), anti-LC3 (Sigma, L8918), anti-p62 (CST, 5114), anti-GAPDH (Abclonal, Wuhan, China, AC002), anti-p-AKT (CST, Boston, MA, USA, 4060), anti-AKT (CST, 4685), anti-Myh7 (Santa, Dallas, TX, USA, sc-53090), anti-HRP conjugated goat anti-rabbit IgG polyclonal Ab (HuaBio, Hangzhou, China, HA1001), and anti-HRP conjugated goat anti-mouse IgG polyclonal Ab (HuaBio, HA1006). The immunoreactions were visualized with Super Signal West Pico PLUS (Thermo Fisher Scientific, Waltham, MA, USA, 34577) and imaged by Azure C400 Imaging Systems.

### 4.6. Immunofluorescence

For the immunofluorescence assay, fixed H9C2 cells and primary cardiomyocytes or tissue sections were washed three times with PBS, permeabilized, and blocked using PBS added with 0.1% Triton X-100 and 1% BSA at room temperature. Samples were stained with the following primary antibodies: anti-LC3 (Sigma, L8918), anti-p62 (CST, 5114), anti-α-actinin (Sigma, A7811), goat anti-mouse IgG (H + L) highly cross-adsorbed secondary antibody, Alexa Fluor Plus 555 (diluted 1:400, Thermo Fisher Scientific, A32727), goat anti-rabbit IgG (H + L) highly cross-adsorbed secondary antibody, Alexa Fluor Plus 488 (diluted 1:400, Thermo Fisher Scientific, A32731). Nuclei were stained with DAPI (Sigma, D9542). Images were obtained using a Nikon C2 plus (Nikon, Japan) confocal microscope and measured by using Image J (v1.37) software.

### 4.7. Hematoxylin-Eosin (HE) and Sirius Red Staining

Heart tissues were harvested and immediately fixed in 4% paraformaldehyde for 48 h. Paraffin sections of 5 μm of thickness were prepared. HE and Sirius Red staining were performed by the core facilities of the University School of Medicine in Zhejiang. Images were captured using VS200 (Olympus, Tokyo, Japan) and measured by using Image J (v1.37) software.

### 4.8. Electron Microscope (EM)

Heart tissues were harvested and immediately fixed in glutaric dialdehyde for 48 h at 4 ℃, followed by a fixation with 1% osmium tetroxide, dehydrated with different acetone concentrations, and placed in embedding solution. Sections of 98 nm of thickness were placed on copper grids, and discs were examined with a Talos L120C (Thermo Scientific, USA) electron microscope.

### 4.9. RNA-Sequencing and Bioinformatics Analysis

For RNA-sequencing experiments, 3 control *Vps4a^fl/fl^* mice and 6 *Vps4a^fl/fl^ Myh6* mice were analyzed. In particular, the *Vps4a^fl/fl^ Myh6* mice were divided into two separate groups (mild and severe) based on the degree of dyspnea and a lack of physical activity. Total RNA was extracted with Trizol (Invitrogen, USA) from the hearts of *Vps4a^fl/fl^* and *Vps4a^fl/fl^ Myh6* mice. The RNA quality was confirmed using a Standard Sensitivity RNA Analysis kit (15 nt) (DNF-471, Bio-Thing, Sunnyvale, CA, USA) with a Fragment Analyzer. BGISEQ500 (BGI-Shenzhen, China) was employed for RNA sequencing. Differentially expressed genes (DEGs) were identified by DESeq2 (v1.26.0) with q ≤ 0.05 and fold change ≥1.3. The heatmap was plotted by pheatmap (v1.0.12) according to the DEGs. Then, to gain further insight into the differences between *Vps4a^fl/fl^* and *Vps4a^fl/fl^ Myh6* mice in biological processes, cellular components, molecular functions, and signaling pathways, GO and KEGG enrichment analysis was conducted using cluster Profiler (3.14.3). The significant levels of terms and pathways were corrected by *p*-value with a threshold (*p*-value < 0.05, Bonferroni corrected). The results of GO analysis and KEGG analysis are drawn by ggplot2 (3.3.6).

### 4.10. Echocardiography

To assess the cardiac contractile function, mice were anesthetized with 2% isoflurane and then subjected to transthoracic echocardiography using a Vevo2100 system (Visual Sonics, Toronto, ON, Canada). M-mode recordings were captured and analyzed to assess the left ventricular ejection fraction (EF), left ventricular internal diameter end-diastole (LVIDd), and left ventricular internal diameter end-systole (LVIDs). Ventricular fractional shortening (FS) was calculated as follows: (LVIDd-LVIDs)/LVIDd.

### 4.11. Statistical Analysis

Data are represented as mean ± s.e.m. Statistical analyses were performed with Graph Pad Prism (v7.0.0). Two groups with normal distributions were compared by using the unpaired two-tailed Student’s *t*-tests, while two-way ANOVA with Tukey’s multiple comparisons test was used to investigate the statistical significance for multiple group comparisons with two variables. * *p* < 0.05 was considered statistically significant.

## Data Availability

The data presented in the study are available from the corresponding authors.

## Supplementary Materials

The following supporting information can be downloaded at: https://www.mdpi.com/article/10.3390/ijms241310800/s1.
