# Supplementary material for: Vps4a Regulates Autophagic Flux to Prevent Hypertrophic Cardiomyopathy
Source: Int J Mol Sci. 2023 Jun 28;24(13):10800. doi: 10.3390/ijms241310800 (PMC10341959; doi:10.3390/ijms241310800)
Supplement: Supplementary file 1 [file ijms-24-10800-s001.zip › ijms-2427829-supplementary.pdf]

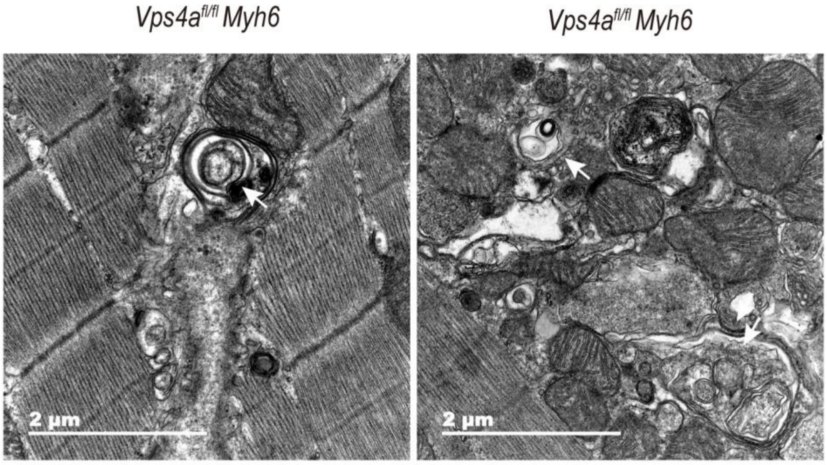

Figure S1. Images of autophagosome in *Vps4a*$^{fl/fl}$ *Myh6* knockout mice observed by electron microscope.

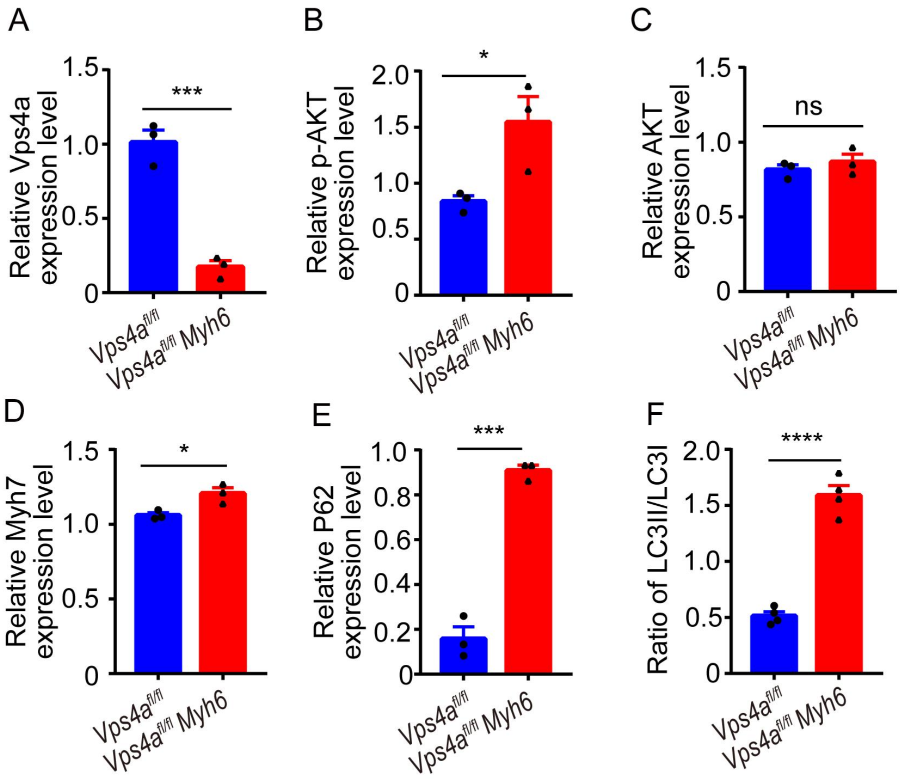

Figure S2. Western blot analysis was performed to measure Vps4a(A), p-AKT(B), AKT(C), Myh7(D) P62(E) and LC3II/LC3I(F) protein expression. n.s., not significant, * p<0.05, *** p<0.001, **** p<0.0001. Significant differences between groups as determined by a two-tailed paired Student's t-test in three independent experiments . Each value is presented as the mean ± s. e. m.

Table S1. The date of transthoracic echocardiography for *Vps4a*$^{fl/fl}$ and *Vps4a*$^{fl/fl}$ *myh6* mice.

Measurement	Units	*Vps4a*$^{fl/fl}$ (n=5)	*Vps4a*$^{fl/fl}$ *Myh6* (n=5)
EF	%	67.77 ± 2.467	37.49 ± 3.072
FS	%	37.02 ± 1.696	18.04 ± 1.709
LV Mass	mg	194.1 ± 16.28	209.2 ± 56.44
LV Vol;d	ul	57.48 ± 13.17	88.2 ± 3.837
LV Vol;s	ul	19.52 ± 5.493	54.95 ± 2.898
IVS;d	mm	1.457 ± 0.1883	1.364 ± 0.3257
IVS;s	mm	2.049 ± 0.1336	1.639 ± 0.378
LVID;d	mm	3.601 ± 0.3377	4.407 ± 0.08374
LVID;s	mm	2.286 ± 0.2628	3.61 ± 0.07966
LVPW;d	mm	1.084 ± 0.2124	0.7196 ± 0.06973
LVPW;s	mm	1.333 ± 0.178	0.8014 ± 0.04151

Table S2. The expression level of autophagy-related gene in *Vps4a*$^{fl/fl}$ and *Vps4a*$^{fl/fl}$ *myh6* mice.

Gene	*Vps4a*$^{fl/fl}$			*Vps4a*$^{fl/fl}$ *Myh6* (mild)			*Vps4a*$^{fl/fl}$ *Myh6* (severe)			log2FoldChange	q value
	C4019	C4013	C2269	K4011	K4012	K2266	K2291	K2292	K2293		
Vps4a	992	1113	928	316	207	219	301	279	203	-2.327222	1.72E-41
Atg10	252	274	297	153	192	172	103	140	140	-0.948691	2.85E-05
Atg4d	769	693	1042	572	735	878	495	505	557	-0.882197	1.72E-06
Atg16l2	144	133	136	193	179	168	97	96	120	-0.598043	0.020803
Wdr41	543	567	502	545	524	523	427	480	491	-0.402248	0.012423
Ctsd	14929	15586	15218	28668	23644	22257	31069	28653	28329	0.414255	0.001335
Rab7	3633	3343	4176	4497	3966	4010	6066	5889	5050	0.415997	0.011088
Mtmr3	1112	1301	1184	1638	1496	1512	1966	1805	1741	0.42452	0.00324
Rubcn	754	800	746	1275	1357	1132	663	786	881	0.427747	0.01482
Vps4b	444	479	410	764	667	630	665	714	702	0.447295	0.003605
Tbk1	318	305	263	439	351	368	462	458	492	0.475972	0.007636
Becn1	914	902	905	1669	1177	1200	1478	1547	1449	0.522491	3.99E-05
Hif1a	1279	1131	844	2179	1894	1448	2026	1858	1784	0.605648	0.001503
Dapk1	688	820	642	1140	1087	1077	1407	1171	1177	0.614391	0.00028
Atg101	320	275	349	370	296	365	539	654	471	0.62271	0.0023
Mtor	1115	1187	1032	1423	1479	1413	2236	1873	1750	0.623132	0.000114
Uvrag	372	364	318	493	458	474	661	657	663	0.714967	3.52E-06
Bcl2	154	233	204	718	337	334	293	391	453	0.923784	0.009352
Atg13	1229	1125	870	2089	1648	1578	2951	2678	2649	1.165803	7.88E-13
Bnip3	4391	2556	2015	4682	2302	1920	9442	8243	6978	1.26233	0.006958

Table S3. The expression level of Mitophagy-related gene in *Vps4a*$^{fl/fl}$ and *Vps4a*$^{fl/fl}$ *myh6* mice.

Gene	*Vps4a*$^{fl/fl}$			*Vps4a*$^{fl/f1}$ *Myh6* (mild)			*Vps4a*$^{fl/fl}$ *Myh6* (severe)			log2FoldChange	q value
	C4019	C4013	C2269	K4011	K4012	K2266	K2291	K2292	K2293		
Prkn	93	125.89	116	68	77	74	116	114	94.18	-0.88839	0.003331
Pink1	13402	15085	13879	10327	10542	10300	12105	10987	9862	-0.72463	4.67E-09
Mul1	693	821	1020	703	829	802	680	639	691	-0.52686	0.00373
Hk2	3762	4115	4479	3785	3605	4416	2643	3566	3667	-0.52228	0.000652
Atpif1	1662	2285	2126	1822	1662	1752	1565	1445	1144	-0.49705	0.004774
Optn	1285	1516	1425	1465	1051	1163	1957	1745	1618	-0.49271	0.002578
Hdac6	324	322	283	502	413	434	550	610	488	0.632838	0.000417
Gba	168	190	167	399	327	325	396	402	361	0.711799	0.000201
